# Comparing an in‐person workshop and a postal Delphi survey for involving health service users in health care and health research prioritization

**DOI:** 10.1111/hex.13646

**Published:** 2022-11-08

**Authors:** Lisa Ann Baumann, Anna L. Brütt

**Affiliations:** ^1^ Department of Health Services Research Carl von Ossietzky University of Oldenburg Oldenburg Germany

**Keywords:** participation, PPI, priority setting, rehabilitation

## Abstract

**Introduction:**

To involve health service users in health care and health research priority setting, different methods exist. Which method is most suitable under which circumstances is unknown. We compared a postal Delphi survey and an in‐person workshop to involve health service users in priority settings for rehabilitative care and research in Germany.

**Methods:**

One hundred and eighty‐four former rehabilitants were randomly assigned to a postal Delphi survey (*n* = 152) or an in‐person workshop (*n* = 32). Two hundred and seventy‐six employees in rehabilitation were also invited to the Delphi Survey. The methodological comparison refers only to the sample of rehabilitants. Within each method, the participants agreed on the top 10 priorities for practice improvement and research in rehabilitative care. The priorities were compared descriptively. Participants' satisfaction was measured with the Public and Patient Engagement Evaluation Tool. The usability of both methods was compared based on the effort, time and material costs required for implementation.

**Results:**

Seventy‐five former rehabilitants and 41 employees in rehabilitation completed both Delphi survey rounds. Eleven former rehabilitants participated in the in‐person workshop. Priorities for practice improvement showed a high degree of overlap between both methods whereas research priorities differed greatly. Participants of the in‐person workshop felt significantly better prepared, more listened to and more likely to feel that different views on the topics were discussed. Participants of the Delphi survey expressed difficulties in understanding all survey questions. The Delphi survey was more elaborate in preparation and implementation but caused lower material costs.

**Conclusion:**

The differences in research priorities between the two methods could be due to the different samples, differences in the individual interests of participants or differences in the prioritization process. In‐person workshops seem to be more appropriate for complex topics, where clarifications of questions and deeper discussions are needed. Delphi surveys seem to be more suitable for easily understandable topics, larger sample sizes and when fewer financial resources are available.

**Patient or Public Contribution:**

The different study phases were supported by employees in rehabilitation and former rehabilitants (e.g., developing study documents, and interpreting results).

## 1 INTRODUCTION

There is consensus among politicians and researchers, that health service users need to be more involved in future healthcare decision‐making and health research.^1^, ^2^, ^3^, ^4^, ^5^, ^6^, ^7^ Health service users are either patients, who currently use health services or citizens, who used or will use health services in the future.^6^, ^8^, ^9^ Involving both groups in healthcare decision‐making and research has been shown to improve the quality and patient‐centeredness of the healthcare system.^5^, ^6^, ^10^, ^11^, ^12^, ^13^, ^14^ However, public and patient involvement (PPI) is not yet the standard of practice in these areas. Health care and research agendas are often still determined without health service users and rather shaped by individual or commercial interests of healthcare providers, funders or politicians.^15^, ^16^, ^17^, ^18^, ^19^ As a result, current health policy and research do not adequately address the interests of citizens and patients.^15^, ^16^, ^20^, ^21^

To make the healthcare system more patient‐centred, health service users need to be involved from the very beginning in setting priorities for healthcare and health research.^1^, ^2^ Priority setting includes the collaborative identification, prioritization and consensus building of and on the most important areas for future practice improvement and research in health care.^22^ For the purposes of this study, areas for practice improvement are defined as areas where direct action is needed to improve health care. Research areas, on the other hand, are areas where unanswered questions exist and need to be answered before practical action can be taken. Only a few examples of prioritization processes for areas for practice improvement in health care are described in the scientific literature.^1^, ^3^ Methods used include surveys, focus groups, citizen juries, consensus conferences, negotiated rule‐making, deliberative pooling or citizen councils.^1^, ^3^ Some countries, like the United Kingdom, have further well‐developed standards and procedures for involving citizens and patients in priority settings for health care improvement. One example is the involvement of citizens and patients in the work of Health Watch. The organization operates at the national and local levels and conducts regular surveys of patients on the state of health care and the potential for improving it. The feedback is used to develop agendas for health policy. However, such priority‐setting processes are usually not comprehensively documented or published as grey literature and are therefore not fully reflected in the scientific literature.^23^ In contrast, several joint prioritization processes are described and published for the field of health research.^1^, ^4^, ^24^, ^25^, ^26^, ^27^, ^28^ Methods used for the prioritization of research questions were, for example, group discussions, voting exercises or consensus meetings.^1^, ^4^ The most frequent method used is the *priority‐setting partnership* of the James Lind Alliance (JLA).^1^, ^4^ The JLA is a nonprofit organization from the United Kingdom which brings together patients, healthcare staff and experts to identify future research priorities.^29^, ^30^ The participants of such priority‐setting partnerships are first asked to name important research questions in their view. For these questions, the state of the evidence is checked. If the research questions are identified to be not sufficiently answered yet, they will be prioritized by the participants in an in‐person workshop and summarized in a research agenda.^29^

So far, there is little evidence on how the method chosen for prioritization affects prioritization or which method is most appropriate under which circumstances.^3^, ^7^, ^24^, ^31^, ^32^ We could not identify any comparative study of methods used for priority setting in health care and only two for health research prioritization. Elliott et al.^33^ compared an in‐person and a wiki‐inspired nominal group technique to identify the top 10 research priorities for chronic kidney disease. They involved patients, caregivers, healthcare providers and politicians. Lavallee et al.^32^ compared online crowd‐voting, an in‐person focus group using the nominal group technique and a two‐round postal Delphi survey to identify research priorities for lower back pain. Only patients were involved. In both studies, no standardized evaluation tool was used for assessing the satisfaction and experience of participants. The ranking of the identified research questions varied between the different methods compared in both studies. Participants were most satisfied with the in‐person format, as they could better express their opinions and contribute meaningfully.

Due to the limited evidence regarding the appropriate use of different prioritization methods, more comparative studies are needed. We, therefore, compared a postal Delphi survey and an in‐person workshop (based on the nominal group technique) to involve health service users in setting priorities for rehabilitative care and research. The two methods represent two different and commonly used approaches for health care and health research prioritization. We compared the methods regarding the identified priorities, participants' satisfaction and the implementation effort.

## 2 METHOD

Within the study, areas for practice improvement and research questions were first identified and afterwards prioritized.^34^ The focus of this paper is on the prioritization phase. For prioritization, participants were randomly assigned to either a postal Delphi survey or an in‐person workshop. From each of the two methods, a top 10 list of priorities for practice improvement and research was created. Afterwards, both methods were evaluated and compared.

Before the study was carried out, it was approved by the responsible Ethics Board (number 2019‐150). The reporting of this study was guided by the reporting guideline for priority setting of health research (REPRISE).^22^

### 2.1 Setting

The study was conducted in the setting of inpatient orthopaedic and psychosomatic medical rehabilitation in Germany. In Germany, patients receive rehabilitative care financed by the Pension Insurance Fund to maintain or restore their social participation and to prevent early retirement. Insured persons can apply for rehabilitation every 4 years to the responsible Pension Insurance Fund, which decides on the approval. If the patient's employment is at considerable risk, the health insurance fund can oblige the patient to apply for rehabilitation (§51 SGB V). After successful approval, rehabilitants stay for approximately 3 weeks (orthopaedic rehabilitation) or 6 weeks (psychosomatic rehabilitation) in an inpatient rehabilitation centre. In our study, we involved rehabilitants, who attended a rehabilitation at one of the three inpatient rehabilitation centres of the German Pension Insurance Oldenburg‐Bremen (DRV OL‐HB). The DRV OL‐HB is a regional institution of German Pension Insurance in the north of Germany. The rehabilitation centres of the DRV OL‐HB have a capacity of about 150 beds each and are focused on the treatment of burnout, depression, personality and behavioural disorders or anxiety disorders for psychosomatic rehabilitation and diseases of the musculoskeletal system, related chronic pain and psychosomatic comorbidities for orthopaedic rehabilitation. After rehabilitation, the patient can receive follow‐up care if needed (e.g., outpatient physiotherapy or psychotherapy).^35^

The German rehabilitation sector is already further advanced in terms of patient participation when compared to other healthcare settings.^36^, ^37^ The rehabilitation setting is, for example, the only care setting, where patient participation in rehabilitative care is required by law (German Social Code IX). Rehabilitants must be involved in the planning of their own rehabilitation stay or in the evaluation and quality assurance of rehabilitative services. However, it is not standard yet to involve rehabilitants in priority‐setting processes for rehabilitative care. To our knowledge, this is the first study in Germany, where different approaches to engaging rehabilitants have been used to identify and prioritize areas for practice improvement and research questions in rehabilitation.

### 2.2 Study design

#### 2.2.1 Identifying themes for prioritization

Areas for practice improvement and research questions were identified through a qualitative cross‐sectional survey (paper and online format) between August and November 2020. Former rehabilitants (*n* = 3872) and employees from rehabilitation centres (*n* = 235) and the sociomedical and administration departments of the DRV OL‐HB (*n* = 31) were invited to name relevant areas for practice improvement and research questions for orthopaedic and psychosomatic rehabilitation from their perspective. The response rate for the former rehabilitants was 5.7% (*n* = 217), for the clinicians 13.6% (*n* = 32) and for the employees of the DRV OL‐HB 41.9% (*n* = 13). Survey responses were analysed according to Mayrings qualitative content analysis using an inductively developed coding system.^38^ Because participant responses were rarely related to indication‐specific topics, but primarily to topics that affect rehabilitation as a whole, the responses were analysed collectively. A total of 20 areas for practice improvement and 30 research questions were derived from the responses of the study participants (see Supporting Information: Appendix 1).

#### 2.2.2 Prioritization

##### Delphi survey

The Delphi survey^39^ consisted of two rounds of postal surveys. Participants were asked to assess the importance of each area for practice improvement and each research question on a 5‐point Likert‐type scale (*very important* to *unimportant*). Participant responses from the first Delphi round (June to July 2021) were descriptively analysed. For each topic, the frequency distribution was calculated across the individual response categories and reflected back to the participants in the second Delphi round by using a bar chart. In the second Delphi round (August to September 2021), participants were asked to assess the same areas for practice improvement and research questions again, considering the average rating of all participants. Finally, the areas for practice improvement and research questions were ranked according to their mean score and standard deviation by the research team.

##### In‐person workshop

The 5‐h in‐person workshop was based on the nominal group technique^39^ and included group discussions on the ranking of the areas for practice improvement and research questions. Two weeks before the workshop started, participants received written information material.

The workshop was conducted on a Saturday in September 2021 and started with a short introduction to the process and clarification of questions. Afterwards, the participants were divided into two teams, one dealing with the areas for practice improvement and one with the research questions. The number of areas for practice improvement and research questions was limited to 20 topics each, as the ranking of more topics in the allotted time was considered impractical. If more topics were available from the identification phase, the respective teams made a preselection of the 20 topics to be discussed and ranked at the workshop.

Within the teams, two small groups were formed to rank the topics. Each small group was supervised by a member of the research team. The participants were asked to first rank the topics by their own. Afterwards, the participants discussed their individual rankings with their group. Each small group was given cards with the corresponding topics, which they were to put in order together. In the case that no consent could be achieved on the ranking, adhesive dots were used. Each participant could distribute his points among the topics (more points meant more relevance). The ranks of the topics from the small group ranking were then summarized into an overall team ranking. The results were discussed by the team members and adjusted in case of discrepancies (consent was thought, if this was not possible, dots were used).

The team rankings were finally presented to all workshop participants. All participants had the opportunity to question the team rankings in case of strong discrepancies and to revise it together until all participants agreed on the final rankings.

### 2.3 Study sample and randomization

Former rehabilitants, who already participated in the identification phase and agreed to participate in the prioritization phase as well (*n* = 184) were randomly assigned to one of the two prioritization methods by using the statistical programme R. Thirty‐two former rehabilitants were invited to the in‐person workshop and 152 to the Delphi survey. In addition, 239 employees from the three rehabilitation centres and 37 employees from the DRV OL‐HB were invited to participate in the Delphi survey. We could not involve the rehabilitation employees in the workshop because of ethical considerations. Rehabilitants could have felt uncomfortable with meeting their administrators or formal caregivers. Further, due to the dependency of rehabilitants on their administrators (employees of the DRV OL‐HB decide on the approval of the rehabilitation applications), rehabilitants could also have felt restricted in their participation. In the context of this study, the comparison of the two prioritization methods refers only to the sample of former rehabilitants to rule out any difference between the methods due to the different stakeholders involved.

### 2.4 Evaluation

#### 2.4.1 Differences in prioritization

Differences in the prioritization of areas for practice improvement and research questions by former rehabilitants are described and compared descriptively between the two prioritization methods. We compared the top 10 priorities and recorded the degree of overlap.

#### 2.4.2 Participant satisfaction

To evaluate the experiences of the former rehabilitants, we used the German version of the Public and Patient Engagement Evaluation Tool.^40^ The questionnaire consists of several 5‐point Likert‐type items (1 = *don't agree at all*, 5 = *strongly agree*) and free‐text fields, asking participants about their general participation experience, their satisfaction with their participation and their opinion on the future use of the results. Additionally, we added questions on the agreement with the final rankings. All former rehabilitants, who participated in the prioritization phase were invited to participate in the evaluation survey. The participants of the in‐person workshop filled out the questionnaire directly after the workshop while the participants of the Delphi survey received the evaluation questionnaire together with the ranking results of the second Delphi survey round by post.

#### 2.4.3 Method usability

We assessed the usability of both methods by comparing the implementation effort (necessary steps for preparation), the time and personal resources required for implementation (time of preparation, execution and evaluation) and the approximate material costs.

### 2.5 Statistical analyses

To assess possible differences between the characteristics of the former rehabilitants in both prioritization methods, we used descriptive statistics and calculated a *χ* ^2^ test (or Fisher's exact test, if the expected frequency in the cells was less than 5). To test whether the former rehabilitants rated their satisfaction and experience with the process and the results of the prioritization process significantly different between the two methods, we used the Mann–Whitney *U*‐test. Written comments of the evaluation survey were qualitatively analysed.

The significance level was set on a two‐sided *p*‐value <.05 for all statistical tests. Because we performed multiple tests to identify differences between the groups (for participants' characteristics and participants' satisfaction/experience), we adjusted the *p*‐value using the Bonferroni correction (*p*‐value/number of tests). By this, we accounted for what is called Alpha error accumulation. If multiple tests are performed on the same hypotheses, the greater the probability of obtaining a *p*‐value <.05 and thus obtaining a result that is falsely significant. All statistical analyses were carried out in SPSS Version 26.

### 2.6 Patient and public involvement

The project was accompanied by a project advisory board which was involved in the planning and implementation of the study, the development of study material and the interpretation of the results. The board consisted of three clinicians from the participating rehabilitation centres and three employees from the DRV OL‐HB. We further aimed to include one rehabilitant, but this did not succeed over the entire duration of the project so that a rehabilitant was present in the phases of interpreting the results from the identification phase and in the preparation of the study materials for the prioritization phase. Four rehabilitants were further involved in the pretesting of the questionnaire for the identification phase.

## 3 RESULTS

Of the 152 former rehabilitants who were randomly assigned to the Delphi survey, 92 (response rate: 63%) participated in the first round of the Delphi survey (some letters could not be delivered and are therefore not included in the calculation of the response rates). Seventy‐five (81.5%) of these also participated in the second round. Eleven of the 32 invited former rehabilitants (35.5%) participated in the in‐person workshop. The sociodemographic data of the former rehabilitants who participated in the Delphi survey and the in‐person workshop and the calculation of significant differences between both groups are shown in Table 1. Besides the former rehabilitants, 46 of the 239 invited clinicians (19.3%) and 9 of the 37 invited employees from the DRV OL‐HB (24.3%) participated in the first Delphi round. Of those, 33 clinicians (82.5%) and 8 employees of the DRV OL‐HB (88.9%) participated also in the second survey round.

**Table 1:** Characteristics of former rehabilitants who participated in the Delphi survey and the in‐person workshop

	Participants of Delphi survey (*n* = 75)	Participants of in‐person workshop (*n* = 11)	*χ* ^2^/Fisher statistic
Gender			
Male	46.7%	54.5%	0.20 (*p* = .654)
Female	52%	45.5%	
Missing	1.3%	0%	
Age			
18–29	1.3%	0%	1.80 (*p* = .814)
30–39	4%	0%	
40–49	16%	18.2%	
50–59	54.7%	45.5%	
60–69	24%	36.4%	
Missing	0%	0%	
Education			
Without school‐leaving qualification	1.3%	0%	3.54 (*p* = .518)
Secondary school diploma	80%	63.6%	
Technical baccalaureate/high school diploma	14.7%	36.4%	
University of applied sciences/university	4%	0%	
Missing	0%	0%	
Indication			
Orthopaedic	45.3%	36.4%	0.68 (*p* = .708)
Psychosomatic	33.3%	45.5%	
Both	21.3%	18.2%	
Missing	0%	0%	
Years with disease			
<1	0%	0%	8.66 (*p* = .016)
1–5	22.7%	27.3%	
6–10	16%	54.5%	
11–15	17.3%	0%	
>15	44%	18.2%	
Missing	0%	0%	
Number of rehabilitation stays			
1–2	54.7%	63.6%	4.09 (*p* = .193)
3–4	32%	18.2%	
5–6	9.3%	0%	
>6	4%	18.2%	
Missing	0%	0%	
Satisfaction with own rehabilitation			
Very satisfied	36%	27.3%	**16.35** * **(*p*** = **.001)**
Satisfied	44%	27.3%	
Neither nor	14.7%	9.1%	
Not satisfied	0%	36.4%	
Not satisfied at all	1.3%	0%	
Missing	4%	0%	

*Note*: If the expected frequency in the cells was less than 5, Fisher's exact test was calculated instead of *χ* ^2^. The value in bold are those where a significant difference was found between the groups compared in the study.

* *p* < .007 (Bonferroni adjusted *p*‐value [*p*‐value of .05/7]).

### 3.1 Comparison of the top 10 priorities for practice improvement and research

The identified top 10 priorities for practice improvement and research for orthopaedic and psychosomatic rehabilitation by former rehabilitants for each method are shown in Tables 2 and 3.

**Table 2:** Comparison of top 10 areas for practice improvement identified by former rehabilitants between the in‐person workshop and Delphi survey

Top 10 priorities for practice improvement			
Top 10 in‐person workshop	Rank Delphi survey	Top 10 Delphi survey	Rank in‐person workshop
Implementation of a holistic therapy approach	1	Implementation of a holistic therapy approach	1
Thorough initial examination	3	Individualize rehabilitative treatment	8
Participation of rehabilitants in rehabilitation	5	Thorough initial examination	2
Quality assurance of rehabilitation treatment	9	Preparation of rehabilitation stay	17
Application process for rehabilitation	7	Participation of rehabilitants in rehabilitation	3
Support of rehabilitants following rehabilitation	11	Discharge management	15
Making rehabilitation sustainable	20	Application process for rehabilitation	5
Individualize rehabilitative treatment	2	Patient education	9
Patient education	8	Quality assurance of rehabilitation treatment	4
Re‐organization of the rehabilitation stay	12	Re‐organization of already offered therapies and measures in rehabilitation centres	13

**Table 3:** Comparison of top 10 research questions identified by former rehabilitants between in‐person workshop and Delphi survey

Top 10 priorities for research			
Top 10 in‐person workshop	Rank Delphi survey	Top 10 Delphi survey	Rank in‐person workshop
How do insureds rate access to rehabilitation measures?	24	What influence does the rehabilitant's motivation have on the treatment outcome?	6
What tasks can case managers take on to support the rehabilitant?	26	How can the cooperation between the pension insurance, the healthcare insurance and the employment agency be improved to avoid waiting times and gaps in care?	3
How can the cooperation between the pension insurance, the healthcare insurance and the employment agency be improved to avoid waiting times and gaps in care?	2	How often should rehabilitation be performed to achieve sustainable treatment success?	13
How much time should optimally elapse between an inpatient hospital stay and the subsequent rehabilitation?	12	Which factors are relevant for rehabilitant's satisfaction in rehabilitation?	Excluded during preselection
How do one‐on‐one therapy and group therapy differ in terms of treatment outcome?	22	What influence does the removal of rehabilitants from their home environment have on the treatment outcome?	9
What influence does the rehabilitant's motivation have on the treatment outcome?	1	What are the main factors that determine the success of treatment in rehabilitation?	Excluded during preselection
How can rehabilitation be designed to meet the individual needs of rehabilitants?	20	What influence does the obligation to file an application for rehabilitation in the case of a significant risk to gainful employment (allocation via §51 SGB V) have on the treatment outcome and rehabilitants satisfaction?	Excluded during preselection
How can early steering, especially into psychosomatic rehabilitation, be achieved?	12	How do an endurance‐oriented and a strength‐oriented training programme differ in terms of treatment outcome for back pain?	Excluded during preselection
What influence does the removal of rehabilitants from their home environment have on the treatment outcome?	5	What is the effect of regular rehabilitation (every 4 years) on the course of illness and ability to work?	16
How can the effectiveness of follow‐up services following rehabilitation be increased?	19	How long should rehabilitation ideally last?	18

A comparison of the ranks for the areas for practice improvement between both methods revealed a large proportion of overlap. Seven areas were present in both top 10. The first rank was the same for both methods (‘Implementation of a holistic therapy approach’) and only a few areas differed considerably in their ranks. In both rankings, it gets clear that participants saw a need for more individualized rehabilitative interventions as well as increased education and participation of rehabilitants about and in their rehabilitation.

In contrast, the priorities for research differed clearly between the two methods. Only three research questions were present in both top 10 and the difference between the ranks was quite high for most of the questions. The former rehabilitants in the in‐person workshop saw a priority need for research in the analysis and further development of the rehabilitation system with a focus on access to rehabilitation and the support of rehabilitants during and after rehabilitation. Former rehabilitants in the Delphi survey also saw a need for research regarding the analysis and further development of the rehabilitation system, but with a focus on structural issues such as the duration and frequency of a rehabilitation stay. Further, questions regarding the theoretical and methodological foundation of rehabilitation (e.g., relevant factors for patient satisfaction) were more relevant to the former rehabilitants in the Delphi survey.

### 3.2 Participants satisfaction

Fifty‐four former rehabilitants from the Delphi survey (response rate: 72%) and all former rehabilitants from the in‐person workshop (*n* = 11) participated in the evaluation survey. The sociodemographic characteristics of the participants can be found in Table 4.

**Table 4:** Sociodemographic data of participants in evaluation survey

	Participants of Delphi survey (*n* = 54)	Participants of in‐person workshop (*n* = 11)
Gender		
Male	46.3%	54.5%
Female	51.9%	45.5%
Missing	1.9%	0%
Age		
18–29	0%	0%
30–39	3.7%	0%
40–49	20.4%	18.2%
50–59	53.7%	45.5%
60–69	22.2%	36.4%
Missing	0%	0%
Education		
Without school‐leaving qualification	1.9%	0%
Secondary school diploma	75.9%	63.7%
Technical baccalaureate/high school diploma	16.7%	36.4%
University of applied sciences/university	5.6%	0%
Missing	0%	0%
Indication		
Orthopaedic	44.4%	36.4%
Psychosomatic	33.3%	45.5%
Both	22.2%	18.2%
Missing	0%	0%
Years with disease		
<1	0%	0%
1–5	24.1%	27.3%
6–10	16.7%	54.5%
11–15	16.7%	0%
>15	42.6%	18.2%
Missing	0%	0%
Number of rehabilitations attended		
1–2	55.6%	63.6%
3–4	33.3%	18.2%
5–6	5.6%	0%
>6	5.6%	18.2%
Missing	0%	0%
Satisfaction with own rehabilitation		
Very satisfied	37%	27.3%
Satisfied	48.1%	27.3%
Neither nor	11.1%	9.1%
Not satisfied	0%	36.4%
Not satisfied at all	0%	0%
Missing	3.7%	0%

In both groups, most of the former rehabilitants (strongly) agreed with all statements. The statistical results of the pairwise comparisons between both groups are summarized in Table 5. There was no significant difference in the overall satisfaction with the priority‐setting exercise as well as the satisfaction with the final results. However, former rehabilitants from the in‐person workshop significantly felt better prepared for the discussion on the topics (*Median workshop* = 5, *Median Delphi* = 4, *U* = 163.5, *p* = .002, *moderate effect* [*r* = .38]) and more heard in their views (*Median workshop* = 5, *Median Delphi* = 4, *U* = 155.5, *p* = .001, *moderate effect* [*r* = .40]).^41^ The workshop participants further significantly more agreed, that the participants represented different perspectives on the topics discussed (*Median workshop* = 5, *Median Delphi* = 4, *U* = 125.5, *p* = .001, *moderate effect* [*r* = .44]) and that a range of views on the topics discussed were shared (*Median workshop* = 5, *Median Delphi* = 4, *U* = 132.5, *p* = .001, *moderate effect* [*r* = .41]).

**Table 5:** Statistical results of the evaluation study

Statements in evaluation survey	Median Delphi (IQR)	Median workshop (IQR)	Mann–Whitney *U* (*p‐*value)	*z*	*r*
I had a clear understanding of the purpose of the Delphi survey/workshop.	4 (0)	4 (1)	258.0 (*p* = .385)	−0.869	
The supports I needed to participate were available.	4 (0)	4 (1)	271.5 (*p* = .562)	−0.580	
I had enough information to contribute to the topic being discussed.	4 (0)	5 (1)	**163.5** * (***p*** = **.002**)	−3.042	.38
I was able to express my views freely.	4 (1)	5 (1)	197.5 (*p* = .059)	−1.886	
I feel that my views were heard.	4 (0)	5 (1)	**155.5** * (***p*** = **.001)**	−3.220	.40
A wide range of views on the topic discussed was shared.	4 (0)	5 (1)	**132.5** * (***p*** = **.001)**	−3.341	.41
The individuals participating in the Delphi survey/workshop represented a broad range of perspectives on the topic.	4 (0)	5 (1)	**128.5** * (***p*** = **.001)**	−3.457	.44
I think that the Delphi survey/workshop achieved its objectives.	4 (0)	5 (1)	167.5 (*p* = .012)	−2.516	.32
I am confident the input provided through the Delphi survey/workshop will be used by the German Pension Insurance Oldenburg‐Bremen.	4 (0)	4 (1)	272.0 (*p* = .753)	−0.315	
I think the input provided through the Delphi survey/workshop will make a difference in the work of the research group and the German Pension Insurance Oldenburg‐Bremen.	4 (0)	4 (1)	250.0 (*p* = .427)	−0.794	
As a result of my participation in the Delphi survey/workshop, I am better informed about the need for practice improvement and research in rehabilitation.	4 (0)	4 (1)	224.5 (*p* = .098)	−1.652	
Overall, I was satisfied with this Delphi survey/workshop.	4 (0)	4 (1)	190.5 (*p* = .024)	−2.260	.28
This Delphi survey/workshop was a good use of my time.	4 (1)	5 (1)	216.0 (*p* = .123)	−1.541	
I agree with the list created for the priority areas for practice improvement.	4 (1)	4 (1)	207.0 (*p* = .088)	−1.704	
I agree with the list created for the priority areas for research.	4 (1)	5 (1)	187.0 (*p* = .044)	−2.011	.25
I am satisfied with the results of the Delphi survey/workshop.	4 (0)	5 (1)	175.5 (*p* = .014)	−2.458	.30

*Note*: Effect size was only calculated for significant differences. The values in bold are those where a significant difference was found between the groups compared in the study.

Abbreviations: IQR, interquartile range; *r*, Pearson correlation coefficient.

* *p* < .003 (Bonferroni adjusted *p*‐value [*p*‐value of .05/16]).

The analysis of the written comments showed, that some Delphi survey participants had difficulties in understanding and answering the survey questions, especially in the first Delphi round.

### 3.3 Methods usability

Detailed information on the usability of both methods is summarized in Table 6. The effort for the preparation, implementation and analysis of the results was higher for the Delphi survey. The in‐person workshop was also time‐consuming in the preparatory phase, but directly usable results were available afterwards.

**Table 6:** Compare of usability for in‐person workshop and Delphi survey

	In‐person workshop	Delphi survey
Effort for preparation and implementation	Development and dispatch of information material Organization of catering Development of hygiene concept (necessary because of Covid‐19 pandemic) Organization of materials (cards with research questions and areas for practical improvement for sorting, moderation materials like colourful cates, glue dots)	Development and dispatch of three questionnaires Manual transfer of participants responses into a digital data format by one person and controlled by a second person Initial analysis of first‐round responses and analysis of final results after second round
Time frame	2 months (planning and preparation in August, Implementation and analysis of results in September)	8 months (preparation from April to June, first survey round from June to July, analysis of initial analysis in July, second survey round from August to September, final analysis in October, evaluation survey in November)
Approximate personal resources	One person (research associate) with 30 h/week for 2 months and four persons (one research associate, one academic researcher, two student assistants) for 9 h each at the workshop day	One person (research associate) with 30 h/week for 8 months and one person (student assistant) with 9 h/week for 1 month
Approximate material costs	Compensation for participants: 100€ per person, in total 1100€ Catering for 11 participants: 561€ Printing information material: 50€ Moderation material: 32€ Letter postage for sending and return for date request and workshop information material: 155€ Envelopes for sending study material to participants: 11€ Printed return envelopes: 5€	Compensation for participants: none Printing questionnaires (2 Delphi rounds and evaluation survey): 513€ Letter postage for sending and return of study documents to former rehabilitants: 671€ Envelopes for sending study material to former rehabilitants: 48€ Printed return envelopes: 22€
	*Total*: 1898€	*Total*: 1254€

*Note*: Only the largest cost items are included in the costs, smaller amounts are not listed, costs were rounded on full euro amounts.

From the point of view of the participants, however, the effort was reversed. Here, the Delphi survey took significantly less time than the in‐person workshop, for which, including travel to and from the workshop, more than half a day had to be taken. The Delphi survey, on the other hand, took about 30–40 min per survey round and could also be completed flexibly in terms of time (within the specified return period).

The in‐person workshop further incurred higher material costs due to the expense of catering and compensation for participants.

## 4 DISCUSSION

We conducted a comparative study of two methods used in health care and health research prioritization. Our study provides important insights into how different prioritization methods work and when to use which method. In the following, we first look at the differences in question prioritization between both methods. Afterwards, participants' satisfaction and experience and the usability of both methods are discussed. Finally, further research need is outlined.

### 4.1 Comparison of the top 10 priorities for practice improvement and research

The differences we observed in the ranking of research questions between different prioritization methods confirm the results identified in the studies of Elliott et al.^33^ and Lavallee et al.^32^ for the setting of rehabilitative care. The first, apparent explanation for the prioritization differences between the two prioritization methods in our study is the different samples in the methods. Besides former rehabilitants, also rehabilitation employees participated in the Delphi survey (with a share of the total Delphi sample of 37% in the first survey round and 35% in the second survey round). Even when we only compared the final rankings of the former rehabilitants, the prioritization of the former rehabilitants, who participated in the Delphi survey, could have been influenced by the opinions of the clinicians and employees of the DRV OL‐HB (after the first Delphi survey round, participants could change their first assessment of each topic after considering the average assessment of all participants).

In the studies of Elliott et al. and Lavallee et al., it was further unclear whether the differences in prioritization were due to the different priority‐setting procedures or to the different characteristics of study participants. By randomly assigning the former rehabilitants to one of the two prioritization methods, we tried to control for sociodemographic differences between the two study samples we compared in our study. However, we identified that our study participants differed significantly in their satisfaction with the rehabilitation they received, with workshop participants less satisfied. This could be due to the randomization process itself, which may have randomly resulted in unequal samples. It could also be due to self‐selection by participants. Individuals, who were more dissatisfied with their rehabilitation stay, might have been more likely to attend the in‐person workshop to take advantage of the opportunity to share personal experiences with other rehabilitants and researchers in the field face‐to‐face. The difference between the groups in satisfaction with their own rehabilitation may have led to the different research priorities in the two methods.

However, because only the prioritization of research questions differed greatly between the two methods (if the differences were due to differences between the study groups, one would expect this to be the case for both areas, research and practice) and previous study results also found differences in the prioritization of research questions between different methods (despite more comparable study samples), we do not believe that the prioritization differences we observed were only due to differences in our study samples. What might also be important are other individual beliefs and interests that we did not collect within our survey. Compared to areas for practice improvement, where former rehabilitants are likely to draw on similar experiences through their rehabilitation stay, research questions might be more driven by individual interests which rehabilitants do not share. It is equally likely that the process for prioritization mostly influenced the decisions of the former rehabilitants. Previous studies suggest, that individuals are more likely to change their opinions on the relevance of different healthcare topics after face‐to‐face discussions.^32^, ^33^, ^42^ Especially in the case of research questions, which are initially more difficult to understand than areas for practice improvement, a face‐to‐face conversation might have had a major impact on ranking decisions. Therefore, the method for prioritization must be carefully selected for the appropriate purpose.

### 4.2 Comparison of participants' satisfaction

As part of our study, we compared participant satisfaction between two prioritization methods using a standardized assessment tool for the first time. We identified that average participant satisfaction was high in both groups (no significant differences), differing only in the range of *satisfied* and *very satisfied*. However, workshop participants rated their experience in some points significantly better. They felt better prepared and listened to, and were more likely to feel that different opinions on the issues were discussed. These results are in line with previous study findings.^3^, ^32^, ^33^ It might reflect that participants of face‐to‐face methods value the personal exchange of different views with other participants and the opportunity for a direct and deeper discussion on the issue. Participants of the in‐person workshop were able to respond directly to what other participants were saying, which could have led to the feeling of being heard more and hearing different opinions on the topics. The workshop participants further received an introduction to the research project and the identified priorities for practice improvement and research. In the course of this, the opportunity of clarifying questions on the course and content of the workshop existed. This may have resulted in workshop participants feeling more informed about the topics discussed, while former rehabilitants who participated in the Delphi survey expressed some difficulties in understanding the questions. Even when the possibility to contact the research team in case of upcoming questions during answering the Delphi survey existed—this was associated with effort and time delays in filling in the survey for the former rehabilitants and was not comparable with a real‐time face‐to‐face discussion.

In conclusion, in‐person workshops seem to be preferred by participants and might also lead to more reliable results as it can be ensured that the task and discussed topics are understood correctly by the participants.^33^ Considering this, an in‐person workshop might be more suitable for complicated topics where the opportunity for direct clarification of questions is needed whether a Delphi survey could be used when the topics for discussion are already comprehensively known by participants.

### 4.3 Comparison of method usability

Advantages in the usability of the in‐person workshop were the lower effort, fewer personal resources and shorter time needed for preparation and implementation when compared to the Delphi survey. Therefore, an in‐person workshop could be especially suitable, when results need to be available quickly (e.g., in health policy decision‐making) and when few personal resources are available.

The material costs for the in‐person workshop were higher than for the Delphi survey. In the literature review of Mitton et al.,^3^ face‐to‐face interventions were also associated with higher costs. Material costs certainly depend on the number of participants involved. But if the number of participants in both methods would have been the same, the material costs for the in‐person workshop would still have been higher due to the costs for catering and participant compensation. A Delphi survey might therefore be more suitable when fewer financial resources are available.

Besides the higher resource need, the in‐person workshop we conducted had further disadvantages. It was held at a specific time and place and was therefore not feasible for some individuals due to work or personal commitments or for individuals with limited mobility. Some of the invited former rehabilitants couldn't participate because the date or time did not suit them. This could lead to a selection bias of participants and might limit the generalizability and expressiveness of the results. Such problems may be solved by conducting the workshop as an online meeting or by conducting an asynchronous discussion among participants on an online platform. However, when implementing the workshop as an online meeting, this could cause other barriers to participation, as access to the internet and a computer is required. An asynchronous discussion, which allows more time flexibility for the participants, on the one hand, makes a moderated and controlled discussion on the other hand more difficult and requires more time.

Another problem of our in‐person workshop might be the ranking procedure, which was mainly based on face‐to‐face discussions between participants. This carries the risk that a few individuals dominate the discussion, which would lead to the unequal representation of individual opinions in the results. The nominal group technique, which guided the methodology of our workshop, has been designed to minimize this risk by having individual and group work phases and involving each participant in the discussion. Within our evaluation survey, we could not find evidence that some individuals felt, that their opinion was insufficiently considered in the final ranking. However, it still remains a risk when a discussion between participants is used for setting priorities.

Where the workshop showed problems, the strengths of the postal Delphi survey became clear. The Delphi survey could be filled in by participants at a convenient time for them within the survey phase. It further saved travel time, which might be especially important when participants from different geographical regions or nonmobile individuals should be involved (this might also be solved by the implementation of the workshop as an online format). This might lead to higher response rates like was the case in our study. Another strength of the Delphi survey is the possibility to involve a larger sample.

### 4.4 Further research need

As there are still very few studies comparing the influence of different methods on prioritization, more research needs to be conducted on this issue. To assess, what difference in prioritization can be attributed to the method itself and what different characteristics of study participants, the same method can be conducted for the same topics but with different participants. To assess the influence of relevant contextual factors (e.g., country, health care setting), (quasi‐)experimental study designs are necessary, where the same method is implemented under different circumstances.^1^, ^33^, ^43^

## 5 LIMITATIONS

Our results should be interpreted considering some limitations. First, we were not able to involve clinicians and employees from the DRV OL‐HB in the in‐person workshop due to ethical considerations. This limited the comparison between both methods since it is more difficult to understand to what extent differences in prioritization are due to differences in the methods or the samples.

Second, our results are limited in their generalization due to the small number of participants. Especially at the in‐person workshop, we had fewer participants than we would have liked. The date of the workshop was not suitable for everyone and there were still contact restrictions because of the Covid‐19 pandemic, so it was also not possible to involve a much larger sample. However, as our results are mostly in line with previous study findings, we are confident that they are at least partly generalizable.

Third, we were not able to fully assess the representativeness of our study sample in the prioritization phase. Ideally, the characteristics of the study participants could have been compared to the entire sample to better assess, if differences in the participant characteristics are caused by participants' self‐selection. However, because the first study phase was conducted anonymously, it was not possible to link the sociodemographic data to the contact information of those participants, who agreed to participate in the second phase of our study.

Fourth, since some Delphi survey participants expressed difficulties in understanding the questions/tasks in the first survey round, it would have been useful to pilot the questionnaire before the survey. We involved only one rehabilitant in the preparation of the study material for the prioritization phase, which may not have been sufficient retrospectively. This should also be considered when assessing the usability of both methods, as an extensive pilot phase of the questionnaire would increase the effort required for a Delphi survey.

Finally, the method we used to rank the topics within the Delphi survey had an influence on our results. We used the mean and standard deviation calculated from participants' assessments, as this is the most commonly used method in the literature.^44^, ^45^, ^46^, ^47^, ^48^, ^49^, ^50^ However, a standardized approach for ranking is missing and the topics could have also been ranked based on the median or percentage of people who rated the topic as very important or important.^51^, ^52^ We tested different methods to rank the topics before we decided on the mean and standard deviation as the decisive factors. The methods resulted in approximately the same top 10, but different subject rankings. Thus, for selecting future priorities and for comparing priorities between different methods, it seems important not to focus too much on the exact ranking, but to consider all of the top 10 issues.

## 6 CONCLUSION

Priorities for practice improvement differed slightly between the in‐person workshop and the Delphi survey. Large differences in the prioritization were observed for research questions. The former rehabilitants who participated in the in‐person workshop rated their experience significantly better regarding their preparation, the feeling of having been heard and the opinion that different views were expressed. Former rehabilitants who participated in the Delphi survey mentioned difficulties in understanding the survey questions. While considering this and the comparison of the usability of both methods, in‐person workshops seem to be more appropriate for complex topics and issues on which results need to be available quickly and when few personal resources are available. Delphi surveys instead are more suitable for easily understandable topics, for involving a larger sample and when fewer financial resources are available.

Our results can help organizers of prioritization events with health service users to decide, which method is most appropriate for their purposes and circumstances. More studies comparing different methods for priority setting in health care and health research are needed to develop clear recommendations on when to use which method.^3^, ^7^, ^24^, ^31^, ^32^

## AUTHOR CONTRIBUTIONS

Anna L. Brütt contributed to the development of the study design. Lisa Ann Baumann and Anna L. Brütt contributed to the development of the Delphi survey questionnaire and planned and conducted the in‐person workshop. Lisa Ann Baumann did the statistical analyses and wrote the manuscript. Anna L. Brütt supervised the process of manuscript preparation and edited the manuscript. Both authors approved the final version of the manuscript.

## CONFLICT OF INTEREST

The authors declare no conflict of interest.

## Supporting information

Supporting information.

## Data Availability

The data that support the findings of this study are available from the corresponding author upon reasonable request.
