# Supplementary material for: Comparing an in‐person workshop and a postal Delphi survey for involving health service users in health care and health research prioritization
Source: Health Expect. 2022 Nov 8;26(1):199–212. doi: 10.1111/hex.13646 (PMC9854299; doi:10.1111/hex.13646)
Supplement: Supplementary file 1 — Supporting information. [file HEX-26--s001.docx]

**Appendix 1**

**Identified areas for practise improvement in orthopaedic and psychosomatic rehabilitation**

| **Area for practise improvement** | **Content** |
| --- | --- |
| **before rehabilitation** | |
| Preperation of rehabilitation stay | - Clarify symptoms and complaints of rehabilitants before the rehabilitation stay sufficiently - Inform rehabilitants about the goals and purpose of rehabilitation and the process of rehabilitation treatment before the start of rehabilitation - Only admit motivated rehabilitants to rehabilitation |
| **during rehabilitation** | |
| Implementation of a holistic therapy approach | - Take a look at several clinical pictures and causes - Consider psychological and physical problems together |
| Individualize rehabilitative treatment | - Adapt the rehabilitation treatment specifically to the individual needs and clinical pictures of the individual rehabilitants |
| Thorough initial examination | - Allow sufficient time for the initial examination - Perform comprehensive diagnostics at the beginning of rehabilitation |
| Discharge management | - Formulate discharge letter precisely - Consider different sources for the socio-medical assessment and evaluation of the rehabilitant’s ability to work |
| Offer of further therapies and measures in rehabilitation | - Expand therapeutic offerings in rehabilitation clinics - Expand leisure activities in the rehabilitation clinics - Implement target group-specific offers (e.g. for shift workers or rehabilitants with burnout) |
| Re-organization of already offered therapies and measures in rehabilitation centres | - Offer more individual therapy - Compose groups in a more targeted way and reduce group size - Adapt information sessions more specifically to rehabilitants needs |
| Re-organization of the rehabilitation stay | - Change creation and adjustment of rehabilitation plan (e.g. individual adjustment of number of treatments, more time between treatments, regular adjustment of plan during stay) - Ensure access to rehabilitation courses/avoid booking out - Avoid change of therapist - Avoid therapy cancellation or idle time (e.g. on weekends/holidays) - Change the nutritional offer in the rehabilitation clinics |
| Joint togetherness during the rehabilitation stay | - Ensure very good German language skills of clinic staff - Prepare staff for different language skills of rehabilitants - deal with rehabilitants in a friendly and emphatic manner - Manage conflicts between rehabilitants |
| Patient education | - Improve rehabilitant’s education about their disease, different treatment options, and strategies for coping with the disease - Strengthen the guidance of rehabilitants in the implementation of therapy |
| Participation of rehabilitants in rehabilitation | - Involve rehabilitants in the selection of a clinic and the development of a therapy plan - Take rehabilitants comments, problems and fears seriously. |
| **after rehabilitation** | |
| Support of rehabilitants following rehabilitation | - Supporting rehabilitants in their search for and use of further health care services and facilities - Expand social legal counseling - Provide aids suitable for (everyday) use - Expand aftercare services - Offer and extend aftercare digitally |
| Making rehabilitation sustainable | - Involve the rehabilitant’s environment in the rehabilitation treatment - Support rehabilitants in the transfer of exercises and knowledge from rehabilitation to everyday life - Follow up rehabilitant’s participation in further measures after rehabilitation |
| **concerning the whole rehabilitation** | |
| Measurement of treatment success and satisfaction of rehabilitants in rehabilitation | - Measure the short- and long-term success of treatment in rehabilitation - Check the benefit of therapies and measures - Rehabilitants evaluate their rehabilitation afterwards |
| Working in the rehabilitation team | - Strengthening cooperation and exchange between the individual professional groups in the rehabilitation clinic - Valuing employees |
| Cooperation in the rehabilitation system | - Intensify cooperation between rehabilitation clinics and other service providers (e.g., family doctors) - Intensify cooperation between the German Pension Insurance Fund and other social insurance agencies, pension offices and employers - Strengthen exchange between the German Pension Insurance Fund and rehabilitation clinics |
| Application process for rehabilitation | - Ability to customize the start, duration and frequency of rehabilitation - Reduce waiting times for a rehabilitation place - Simplify the application process for rehabilitation and involve a physician in the application process |
| Quality assurance of rehabilitation treatment | - Make rehabilitation therapy standards (RTS) and KTL specifications more flexible - Avoid drug shortages - Maintain sufficient staff in rehabilitation clinics - Train staff - Provide evidence-based therapies and interventions |
| Spatial and technical equipment of the rehabilitation clinics | - Modernize premises, sports equipment and information materials - Set up community rooms - Set up wifi access |
| Development of rehabilitation concepts for pandemic periods | - Developing concepts of what rehabilitation can look like under pandemic conditions |

**Identified research questions for orthopaedic and psychosomatic rehabilitation**

| **Research uncertainty** |
| --- |
| **Development of rehabilitation interventions** |
| How can rehabilitation be designed to meet the individual needs of rehabilitants? |
| How can the effectiveness of follow-up services following rehabilitation be increased? |
| How can exercises and content in rehabilitation be designed for everyday use? |
| **Evaluation of rehabilitation interventions** |
| What is the benefit of informational lectures during rehabilitation compared to active therapies (such as rehabilitation sports), in terms of treatment outcome? |
| How do open and closed therapy groups differ in terms of treatment outcome and rehabilitant satisfaction? |
| How do an endurance-oriented and a strength-oriented training program differ in terms of treatment outcome for back pain? |
| How do individual therapy and group therapy differ in terms of treatment outcome? |
| What impact did the introduction of the rehabilitation therapy standards have on treatment outcomes? |
| How does a smoking cessation program in orthopedic rehabilitation affect treatment outcomes? |
| Is the aftercare offered following rehabilitation sufficient to ensure that rehabilitants continue their treatment in a timely manner? |
| **Analysis and further development of the rehabilitation system** |
| What influence does the removal of rahabilitants from their home environment have on the treatment outcome? |
| What influence does the obligation to file an application for rehabilitation in the case of a significant risk to gainful employment (allocation via §51 SGB V) have on the treatment outcome and rahabilitants satisfaction? |
| How does a rehabilitants wish for retirement affect the treatment outcome and rehabilitants satisfaction in rehabilitation? |
| How does medical rehabilitation in Germany compare to rehabilitation systems in other countries? |
| What is the effect of regular rehabilitation (every 4 years) on the course of illness and ability to work? |
| What influence does a change of therapist have on the treatment outcome? |
| How can rehabilitation clinics deal with the shortage of skilled workers? |
| How do insureds rate access to rehabilitation measures? |
| How can early steering, especially into psychosomatic rehabilitation, be achieved? |
| How does an additional stay in rehabilitation (for example, 6 months after initial rehabilitation) to consolidate what has been learned affect the long-term success of rehabilitation treatment? |
| How long should rehabilitation ideally last? |
| How often should rehabilitation be performed to achieve sustainable treatment success? |
| How much time should optimally elapse between an inpatient hospital stay and the subsequent rehabilitation? |
| How can the cooperation between the pension insurance, the health care insurance and the employment agency be improved in order to avoid waiting times and gaps in care? |
| How can inpatient rehabilitation treatment be better linked to pre- and post-treatment? |
| What tasks can case managers take on to support rehabilitants? |
| How can rehabilitation be designed in times of a pandemic (e.g. Covid-19)? |
| **Theory and methodological principles in rehabilitation** |
| What are the main factors that determine the success of treatment in rehabilitation? |
| What influence does the rehabilitant’s motivation have on the treatment outcome? |
| Which factors are relevant for rehabilitant’s satisfaction in rehabilitation? |
